# Self-Determination Theory and Accountant Employees’ Psychological Wellbeing: The Roles of Positive Affectivity and Psychological Safety

**DOI:** 10.3389/fpsyg.2022.870771

**Published:** 2022-05-25

**Authors:** Philip Teng Lin, Thinh Truong Vu, Van Phuong Nguyen, Qi Wu

**Affiliations:** ^1^School of Accounting, Guangdong University of Foreign Studies, Guangzhou, China; ^2^Department of Business Administration, Dong Nai Technology University, Bien Hoa, Vietnam; ^3^Department of Economics and Management, Thuyloi University, Hanoi, Vietnam; ^4^Business School, Shantou University, Shantou, China

**Keywords:** self-determination theory, psychological wellbeing, positive affectivity, psychological safety, accountant employees

## Abstract

This study investigates the influence of self-determination motivations on accountant employees’ psychological wellbeing with the mediating role of positive affectivity and the moderating role of psychological safety. Multivariate analysis and structural equation modeling are used to analyze a three-way time-lagged sample data of 391 accountant employees. Results indicate that positive affectivity positively mediates the relationship between extrinsic motivation and psychological wellbeing and between intrinsic motivation and psychological wellbeing. Furthermore, psychological safety positively moderates the relationship between extrinsic motivation and positive affectivity and between intrinsic motivation and positive affectivity. In addition, psychological safety also positively moderates the relationship between positive affectivity and psychological wellbeing. The findings of this study provide implications for researchers and business managers in managing and enhancing accountant employees’ psychological wellbeing.

## Introduction

Psychological wellbeing is a global assessment of a person’s quality of life (Diener et al., 1985). It has been an important research topic in the fields of psychology and organizational management (Karapinar et al., 2020). Prior studies have reported that psychological wellbeing is an important antecedent of job performance (Wright and Cropanzano, 2000), health (Cartwright and Cooper, 2008), positive job and work attitudes (Robertson et al., 2012), organizational performance (Loon et al., 2019), and employee performance (Boudrias et al., 2021). As psychological wellbeing plays an important role in influencing employees’ motivations, emotions, and behaviors at work and in social life, researchers and business managers have focused on investigating the antecedents of psychological wellbeing with an effort to improve employees’ psychological wellbeing (Karapinar et al., 2020; Kundi et al., 2020; Boudrias et al., 2021).

This study has conducted a systematic review of research on the antecedents of employees’ psychological wellbeing in the psychology, human resource management, and organizational management literature. As shown in Table 1, several research gaps exist in the current literature. First, although several predictors of psychological wellbeing have been investigated in prior studies, including psychological interventions, personality, digital media use, and human resource practices (Ang et al., 2017; Anglim et al., 2020; Twenge and Martin, 2020; Koydemir et al., 2021), the prediction of individual factors, such as motivations, has been largely ignored in prior studies. Second, some studies have determined the mediating or moderating mechanism in the link between antecedents and employees’ psychological wellbeing (Ang et al., 2017; Luo and Hancock, 2020). Unfortunately, the role of emotions (i.e., positive affectivity and psychological safety) has not been examined in the link between individual factors and employees’ psychological wellbeing. Third, most of the prior studies have used the sample data of employees from different industries in different countries. Surprisingly, the psychological wellbeing of accountant employees in emerging countries, such as China, has not been investigated in prior literature. These research gaps need to be addressed to enrich extant literature about the antecedents and mediating and/or moderating mechanisms into the link between antecedents and employees’ psychological wellbeing.

**TABLE 1:** Literature review of antecedents of psychological wellbeing.

Studies	Antecedents	Mediator/moderator	Sample
Koydemir et al., 2021	Psychological interventions	None	Meta-analysis of 223 papers
Anglim et al., 2020	Personality	None	Meta-analysis of 334 papers
Twenge and Martin, 2020	Digital media use	Gender	221,096 adolescents in the United States and United Kingdom
Luo and Hancock, 2020	Self-disclosure on social media	Perceived connectedness, social support, motivations	None (Concept paper)
Ang et al., 2017	Human resource practices	Leader-member exchange, social connectedness	419 employees and 162 leaders in Australia
Arnold, 2017	Transformational leadership	None	Review of 40 papers
Baluch, 2017	Human resource practices	None	Case study of 8 social services NPS in the United Kingdom.
Guest, 2017	Human resource management	None	None (Concept paper)
Boreham et al., 2016	Employment conditions	None	1,653 respondents from households in Australia
Heffernan and Dundon, 2016	High performance work system	Perceptions fairness	187 employees in Ireland
Huang et al., 2016	High performance work system	None	451 employees and 50 managers of 50 firms in Taiwan
Mihail and Kloutsiniotis, 2016	High performance work system	Employee-employer exchange relationship	297 clinicians in Greek
Arnold and Walsh, 2015	Customer incivility	Meaningful work, perspective taking, transformational leadership	215 employees from service industry in Canada
Decramer et al., 2015	Performance management practices	None	140 nurses in hospitals in Belgium
Boxall and Macky, 2014	High involvement work processes	None	928 employees from Australia and New Zealand
Carvalho and Chambel, 2014	Job characteristics	Work-to-family enrichment	1,390 employees from Portuguese bank
Cañibano, 2013	Innovative human resource management	None	In-depth interviews of 46 employees
Alfes et al., 2012	Human resource practices	Employer trust	613 employees and managers in service firms in the United Kingdom
Böckerman et al., 2012	High involvement management practices	None	3,755 employees in Finland
Kelloway et al., 2012	Transformational leadership	Trust in leadership	705 employees from telecommunication firms in Canada

*n = 20.*

To fill the abovementioned research gaps in the current literature, this study aims to investigate the relationship between self-determination motivations and accountant employees’ psychological wellbeing, with the mediating role of positive affectivity and the moderating role of psychological safety. First, the self-determination theory states two types of motivations, including extrinsic and intrinsic motivations, which have an influence on individuals’ perceptions, emotions, attitudes, and behaviors (Ryan and Deci, 2017). Accordingly, self-determination motivations may trigger accountant employees’ positive affectivity, which in turn enhances their psychological wellbeing (Liang et al., 2018). Unfortunately, the current literature has not determined how self-determination motivations influence employees’ psychological wellbeing. Thus, this study selects self-determination motivations as antecedents of accountant employees’ psychological wellbeing.

Second, positive affectivity refers to the tendency a person experiences the intense pleasant feeling. It is an affective trait that exerts influence on an individual’s perceptions, attitudes, and behaviors (Cropanzano et al., 2003, p. 832). In the workplace, the external incentives from leaders and organizations and internal motivation may have a positive effect on employees’ emotions (Dang and Chou, 2019). That is, employees tend to form a positive psychological state when they receive both internal and external motivations (Babin and Darden, 1996). Consequently, employees may experience a feeling of psychological wellbeing caused by positive affectivity (Cropanzano et al., 2003). Therefore, positive affectivity may play a mediating role in the relationship between self-determination motivations and psychological wellbeing. However, this mediating mechanism has been an absence in the current literature. This leads to the selection of positive affectivity as a mediating variable in the research model of this study.

Third, psychological safety reflects employees’ perception of security and safety in the workplace (Kahn, 1990). This perception may exert an effect on an individual’s motivations, emotions, and behaviors (Dang et al., 2021). In the context of the workplace, when accountant employees perceive high or low level of safety and security, they may have different motivations and experience a positive or negative emotion and feeling of psychological wellbeing (May et al., 2004). That is, psychological safety may have a moderating effect on the relationship between self-determination motivations and emotion and between emotion and psychological wellbeing. Unfortunately, the moderating effect of psychological safety has been underdetermined in the current literature. Therefore, this study chooses psychological safety as a moderating variable in this study’s research model.

Finally, Bragg (2009) stated that the work of an accountant is often complex in nature and differs from other works in an organization. In particular, accountant employees often face a large amount of workload and they have to deal with high pressure and stress in their daily work. This is because accountant employees’ work is often related to statistics, numbers, and mathematics that require a high level of accuracy and quality (Kirkham and Loft, 1993). Furthermore, given the important role of accountant employees and the specific nature of their work in an organization, Syrek et al. (2022) suggested that accountant employees’ wellbeing needs to be cared by leaders and organizations, and it needs more attention from both researchers and business managers to examine the issue of accountant employees’ wellbeing. Therefore, this study takes an advantage of accountant employees as the target research to investigate the relationships among self-determination theory, positive affectivity, psychological safety, and accountant employees’ psychological wellbeing.

## Literature and Hypothesis Development

### Self-Determination Theory

The self-determination theory refers to extrinsic and intrinsic motivation as the two different types of motivation that affect an individual’s emotions, attitudes, and behaviors (Ryan and Deci, 2017). Extrinsic motivation occurs when an individual performs a certain task in order to obtain external incentives and avoid negative outcomes. In contrast, intrinsic motivation is an autonomous form of motivation that occurs when an individual engages in an activity because he or she has a feeling of pleasure and satisfaction from the activity itself (Stupnisky et al., 2018). The self-determination theory has been widely used to explain people’s behavioral outcomes, including job search (Welters et al., 2014), work performance (Howard et al., 2016), work outcomes (Kuvaas et al., 2017), teaching and best practices (Stupnisky et al., 2018), task effort (Liang et al., 2018), and cross-cultural adjustment (Dang and Chou, 2019). Although evidence has shown the effect of self-determination motivations on individuals’ work performance and behavioral outcomes, the influence of self-determination motivations on accountants’ emotions and wellbeing has been an absence in prior literature. This needs to be addressed in this study.

### Positive Affectivity

Positive affectivity refers to a positive psychological state. It is a hedonic tone of emotion in which a person experiences a high pole of pleasant feelings, such as happiness, pleasure, excitement, and enthusiasm (Babin and Darden, 1996). Specifically, Cropanzano et al. (2003) defined the positive affectivity as “the tendency to experience intense pleasant feelings. At the high pole enthusiasm and excitement anchor the dimension of emotion” (Cropanzano et al., 2003, p. 832). Positive affectivity is often associated with positive attitudes and behavioral outcomes in the workplace (Pulligadda et al., 2016; Mohr et al., 2021).

### Psychological Wellbeing

Psychological wellbeing is defined as “individuals’ overall evaluation about their quality of life depending on their standards and emotional experience” (Karapinar et al., 2020). In particular, psychological wellbeing represents “a good state of psychological functions and the fulfillment of personal potential including personal growth, self-acceptance, autonomy, purpose in life, and positive relationship with others” (Zheng et al., 2015). Psychological wellbeing is a complex and multidimensional construct. Ryff (1989) identified 6 dimensions of psychological wellbeing, namely, self-acceptance, personal growth, purpose in life, positive relations with others, environmental mastery, and autonomy. Diener et al. (1985) developed a measurement scale of general life satisfaction as the core dimension of psychological wellbeing. The authors defined this concept as “the individuals’ subjective and global judgment whether the individual is having satisfaction with their life as a whole.” To focus on the judgment and evaluation of overall satisfaction with life, this study follows Diener et al. (1985) and Carvalho and Chambel (2014) to focus on general life satisfaction as a core variable of psychological wellbeing of accountant employees.

### Self-Determination Motivations, Positive Affectivity, and Psychological Wellbeing

According to the self-determination theory, extrinsic incentives, such as salary, money, bonus, and promotion, can act as a driver to affect an employee’s emotions, attitudes, and behaviors (Ryan and Deci, 2008). As human beings, accountant employees have different needs and demands that need to be satisfied. For example, they seek to secure their jobs, obtain a higher salary, get promotion to a higher position, or gain monetary rewards when they perform their tasks (Dang and Chou, 2019). In other words, accountant employees work toward their goals that enable them to obtain needs satisfaction and lead to positive outcomes (Welters et al., 2014).

Furthermore, Babin and Darden (1996) stated that emotion or affectivity can be either negative or positive. Negative emotion refers to a psychological state of sadness, boredom, and even depression, whereas positive emotion is the state of happiness, pleasure, enthusiasm, and excitement. Negative and positive emotion is often induced by a negative and/or positive stimulus and event (Cropanzano et al., 2003). For example, employees often experience positive feelings and satisfaction when they are admired and obtain support from their supervisors and organizations (Mohr et al., 2021). Therefore, according to the self-determination theory, when accountant employees obtain external incentives, they tend to experience a positive emotion and feel satisfied with their lives because extrinsic motivation acts as a psychological mechanism that fulfills accountant employees’ needs and demands (Dang and Chou, 2019). In this case, accountant employees obtain positive outcomes from extrinsic motivation (i.e., incentives and rewards), and their psychological needs and demands are satisfied by these extrinsic motivations (Kuvaas et al., 2017). Accordingly, it is expected that extrinsic motivation enhances accountant employees’ positive affectivity, which in turn increases their feeling of satisfaction with life. The following hypothesis is developed:

***Hypothesis 1.** Positive affectivity positively mediates the relationship between extrinsic motivation and psychological wellbeing.*

In contrast to extrinsic motivation, intrinsic motivation is a type of autonomous motivation, which reflects the interest and enjoyment of an individual’s inner needs (Stupnisky et al., 2018). In an organization, skill development, self-achievement, fun, and happiness are the main intrinsic motivation of employees when they perform tasks and engage in their jobs (Kuvaas et al., 2017). Intrinsically motivated employees often have inner pursuits for personal growth and development, autonomy, and competence (Liang et al., 2018). In the context of accounting work, when an accountant employee has a strong intrinsic motivation, he or she often feels interested, pleasure, and excited about his or her job and organization. That is, intrinsic motivation is linked with high energy levels and persistence, enthusiasm, and engagement (Kuvaas et al., 2017). In this case, accountant employees experience positive feelings and emotions. Consequently, good feeling and positive affectivity states tend to lead to life satisfaction because their inner needs of autonomy and psychological interests are fulfilled by their inner motivation (Ryan and Deci, 2008). Thus, it is expected that intrinsic motivation enhances accountant employees’ positive affectivity, which increases their psychological wellbeing. The following hypothesis is developed:

***Hypothesis 2.** Positive affectivity positively mediates the relationship between intrinsic motivation and psychological wellbeing.*

### Psychological Safety and Its Moderating Role

Psychological safety is the perception of security and feelings of confidence when an individual exposes to the external environment (Kahn, 1990). In particular, psychological safety refers to “feeling able to show and employ one’s self without fear of negative consequences of self-image, status, or career” (Kahn, 1990, p. 708). That is, psychological safety reflects the feelings of safety without any fear of negative consequences (Dang et al., 2021). It is identified as an important antecedent of an individual’s perceptions, motivations, emotions, attitudes, and behaviors (Newman et al., 2017; Dang et al., 2021).

In the context of an organization, the perceptions of psychological safety influence an employee’s motivations, emotions, and behavior (May et al., 2004). For example, Kahn (1990) reported that psychological safety increases an employee’s work engagement and self-expressive behavior. Edmondson (1999) found that psychological safety enhances employees’ motivation to learn in teamworking. Siemsen et al. (2009) suggested the positive influence of psychological safety on co-workers’ knowledge-sharing behavior. Newman et al. (2017) conducted a systematic review of 83 studies from 1990 to 2015. The authors concluded that prior studies have provided evidence for the effect of psychological safety on employees’ motivations, emotions, attitudes, and behaviors at work and organization.

In the case of accounting work, psychological safety is important for accountant employees because they need to be secured to fulfill their tasks and increase their work performance (Kahn, 1990). A high level of psychological safety implies that accountant employees feel safe and confident. In this case, accountant employees tend to have a strong motivation to engage in their work because they can drive their entire energy and efforts toward their tasks (Edmondson, 1999). At the same time, feelings of confidence and security also increase employees’ positive emotions and satisfaction because psychological safety induces employees’ emotional ability to express themselves and experience feeling of comfort (Newman et al., 2017). In other words, a high level of psychological safety strengthens the relationship among motivations, positive affectivity, and psychological wellbeing. In contrast, a low level of psychological safety indicates that accountant employees perceive high risk and uncertainty in their work and organization (Dang et al., 2021). These employees may have a low motivation and experience negative emotion and satisfaction toward their work and organization because perceptions of insecurity raise employees’ feelings of worry about negative consequences (Das and Varshneya, 2017). That is, employees may worry about bad things will happen to them which leads to employees’ psychological depression, negative emotion, and dissatisfaction (Newman et al., 2017). In this case, a low level of psychological safety may weaken the relationships among employees’ motivations, positive affectivity, and psychological wellbeing. Thus, it is expected that psychological safety will moderate the relationship between motivations (i.e., extrinsic and intrinsic motivation) and positive affectivity and between positive affectivity and psychological wellbeing. Accordingly, the following hypotheses are developed:

***Hypothesis 3.** Psychological safety positively moderates the relationship between extrinsic motivation and positive affectivity.*

***Hypothesis 4.** Psychological safety positively moderates the relationship between intrinsic motivation and positive affectivity.*

***Hypothesis 5.** Psychological safety positively moderates the relationship between positive affectivity and psychological wellbeing.*

**FIGURE 1:**
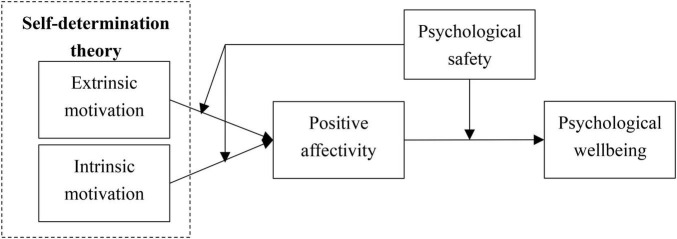
Research model.

The research model is present in Figure 1.

## Methodology

### Measures

#### Independent Variable

This study adopted 10 items from Kuvaas et al. (2017) to measure self-determination motivations. *Extrinsic motivation* was measured with 4 items, namely, (1) if I am supposed to put in extra effort in my job, I need to get extra pay, (2) it is important for me to have an external incentive to strive for in order to do a good job, (3) external incentives, such as bonuses and provisions, are essential for how well I perform my job, and (4) if I had been offered better pay, I would have done a better job. *Intrinsic motivation* was measured with 6 items, namely, (1) the tasks that I do at work are themselves representing a driving power in my job, (2) the tasks that I do at work are enjoyable, (3) my job is meaningful, (4) my job is very exciting, (5) my job is so interesting that it is a motivation in itself, and (6) sometimes, I become so inspired by my job that I almost forget everything else around me. These items were measured using a five-point Likert-type scale (5 = strongly agree, 1 = strong disagree).

#### Dependent Variable

This study measured *psychological wellbeing* using 5 items from Diener et al. (1985), namely, (1) in most ways, my life is close to my ideal, (2) the conditions of my life are excellent, (3) I am satisfied with my life, (4) so far, I have gotten the important things I want in life, and (5) if I could live my life over, I would change almost nothing. These items were measured using a five-point Likert-type scale (5 = strongly agree, 1 = strong disagree).

#### Mediating Variable

*Positive affectivity* was measured using 4 items from Babin and Darden (1996). Respondents were asked to answer the question “To what extend do you feel this way generally at work: (1) happy, (2) pleased, (3) satisfied, and (4) content.” Then, respondents provided their measures from 1 (not at all) to 5 (extremely) for their selection.

#### Moderating Variable

*Psychological safety* was measured using 5 items from Carmeli et al. (2010), namely, (1) I am able to bring up problems and tough issues, (2) people in this organization sometimes reject others for being different, (3) it is safe to take a risk in this organization, (4) it is easy for me to ask other members of this organization for help, and (5) no one in this organization would deliberately act in a way that undermines my efforts. These items were measured using a five-point Likert-type scale (5 = strongly agree, 1 = strongly disagree).

#### Control Variables

The respondents’ characteristics were controlled in the analysis due to their potential impact on the dependent variable, including gender, age, education, income, and marital status (Dang and Chou, 2019).

### Sample Data

This study used a survey questionnaire to collect sample data. The target respondents are accountants working at different companies in Guangdong Province, China. Guangdong is the largest province in China with a population of more than 110 million people. There are several Fortune 500 companies and associated enterprises in Guangdong. This study selected companies in Guangdong as the research sample. We hired a marketing research and human resource consultant company to identify a population list. A total of 500 accountants from 300 companies provided by this consultant company were invited to participate in the survey. The research team used phone calling and face-to-face talking to collect data from the respondents. This study provided monetary incentives to encourage respondents’ willingness and motivation. To avoid the problem of common method bias (CMB), the survey was conducted in three stages. In the first stage (January 2021), the respondents provided measures for the independent variable and personal information. In the second stage (February 2021), the respondents provided measures for the mediating and moderating variables. In the third stage (March 2021), the respondents provided measures for the dependent variable. With the approval of the respondents, the research team recorded the questionnaire process to ensure the consistency of their measures through three stages. We finally received 406 questionnaires, among them 15 questionnaires were invalid with incomplete values. Finally, we collected 391 valid questionnaires with a response rate of 78.2%.

The respondents’ characteristics of the sample data are as follows: (1) gender: 95 men and 296 women; (2) age: 31 respondents were between 20 and 30 years, 348 respondents were between 31 and 40 years, 8 respondents were between 41 and 50 years, and 4 respondents were 51 years or above; (3) education: 358 respondents held an undergraduate degree and 33 respondents held the master’s degree or above; (4) income: 20 respondents had income under US$1,000, 53 respondents had income between US$1,000 and under US$2,000, and 318 respondents had income US$2,000 or above; and (5) marital status: 375 respondents were married and 16 respondents were single.

### Ethical Consideration

This study followed the ethical standard of the China’s National Social Science and APA’s guidance to conduct the survey. Respondents did not need to provide written informed consent because they participated in the survey voluntarily.

### Analysis Methods

Following the studies in the current literature (Carmeli et al., 2010; Kuvaas et al., 2017), this study used SPSS version 24 and SEM-AMOS version 24 to analyze the sample data and test hypotheses. The direct, indirect, and moderating effects were tested using SEM with bootstrap analysis (Preacher et al., 2007; Hair et al., 2010).

## Results

### Measurement Model

As suggested by Hair et al. (2010), data from the survey questionnaire were conducted with a confirmatory factor analysis (CFA). The results of CFA indicate that chi-square/degree of freedom = 489.127/226 = 2.164, comparative fit index (CFI) = 0.925, goodness of fit index (GFI) = 0.926, root mean square error of approximation (RMSEA) = 0.055, indicating a good model fit for a CFA model in this study. Results of this measurement model (CFA) are presented in Table 2.

**TABLE 2:** Results of the measurement model.

Construct	Item	Factor loadings	CR	AVE	Cronbach’s α	Mean	SD
Extrinsic motivation (ExtM)	ExtM1	0.729***	0.82	0.53	0.71	3.85	0.60
	ExtM2	0.757***					
	ExtM3	0.708***					
	ExtM4	0.708***					
Intrinsic motivation (IntM)	IntM1	0.827***	0.89	0.58	0.79	3.88	0.62
	IntM2	0.683***					
	IntM3	0.753***					
	IntM4	0.708***					
	IntM5	0.767***					
	IntM6	0.808***					
Positive affectivity (PosA)	PosA1	0.735***	0.84	0.56	0.71	3.44	0.65
	PosA2	0.754***					
	PosA3	0.755***					
	PosA4	0.762***					
Psychological safety (PsyS)	PsyS1	0.785***	0.86	0.55	0.77	3.54	0.64
	PsyS2	0.791***					
	PsyS3	0.760***					
	PsyS4	0.734***					
	PsyS5	0.621***					
Psychological wellbeing (Well)	Well1	0.746***	0.84	0.52	0.83	3.73	0.61
	Well2	0.720***					
	Well3	0.715***					
	Well4	0.751***					
	Well5	0.673***					

n = 391, ***p < 0.001.

### Reliability and Validity

Table 2 shows the values of Cronbach’s alpha for all variables, namely, 0.71 (extrinsic motivation), 0.79 (intrinsic motivation), 0.71 (positive affectivity), 0.77 (psychological safety), and 0.83 (psychological wellbeing). These values were greater than 0.60 (Hair et al., 2010), indicating good reliability.

Table 2 also shows the values of composite reliability (CR) and average variance extracted (AVE) for all variables, namely, extrinsic motivation (CR = 0.82, AVE = 0.53), intrinsic motivation (CR = 0.89, AVE = 0.58), positive affectivity (CR = 0.86, AVE = 0.56), psychological safety (CR = 0.86, AVE = 0.55), and psychological wellbeing (CR = 0.84, AVE = 0.52). These values were greater than 0.70 (for CR) and 0.50 (for AVE), indicating good convergent reliability.

Table 3 indicates the comparison between square roots of AVE and correlation between variables. It is indicated that all square roots of AVE were greater than all correlations, indicating good discriminant validity. Furthermore, the values of heterotrait-monotrait ratio (HTMT) were all less than 0.85 (Henseler et al., 2015), further confirming the goodness of discriminant validity.

**TABLE 3:** Discriminant validity.

Variable	1	2	3	4	5	VIF
(1) Extrinsic motivation	**0.73**					1.756
(2) Intrinsic motivation	0.53** (0.77)	**0.76**				1.396
(3) Positive affectivity	0.48** (0.68)	0.29** (0.55)	**0.75**			1.488
(4) Psychological safety	0.42** (0.57)	0.22** (0.39)	0.48** (0.64)	**0.74**		1.384
(5) Psychological wellbeing	0.44** (0.58)	0.42** (0.60)	0.46** (0.61)	0.27** (0.79)	**0.72**	
(6) Social desirability	0.01**	−0.09**	−0.07**	0.03**	0.12**	

n = 391, **p < 0.01, the bold values are square roots of AVE, the brackets are HTMT.

### Response Bias, Social Desirability, and Common Method Bias

Response bias was tested by comparing respondents in the first 3 days and those in the last 3 days of the survey. There is no statistical evidence indicating the difference between early respondents and later respondents, implying the non-response bias can be ignored in this study.

Social desirability was tested using five items from Haghighat (2007). Results indicate that the social desirability had a very low correlation with all variables in the research model, namely, extrinsic motivation (*r* = 0.01, *R*^2^ = 0.7%), intrinsic motivation (*r* = –0.09, *R*^2^ = 0.4%), positive affectivity (*r* = –0.07; *R*^2^ = 0.1%), psychological safety (*r* = 0.03, *R*^2^ = 0.2%), and psychological wellbeing (*r* = 0.12, *R*^2^ = 0.1%). Thus, social desirability may not affect the sample data in this study.

The CMB was tested using two different methods. First, a Harman’s one-factor test was conducted. Results of this test indicate that the first factor accounted for 31.45% of the variance, which was less than 50% (Podsakoff et al., 2003). Second, a marker variable test was performed with the variable “blue attitude” (one example: I will buy a blue car). Results indicate that the CMB was less than 1%, which was very small and could not affect the results of hypothesis testing (Griffith and Lusch, 2007). Thus, CMB could be ignored in this study.

### Structural Model

**FIGURE 2:**
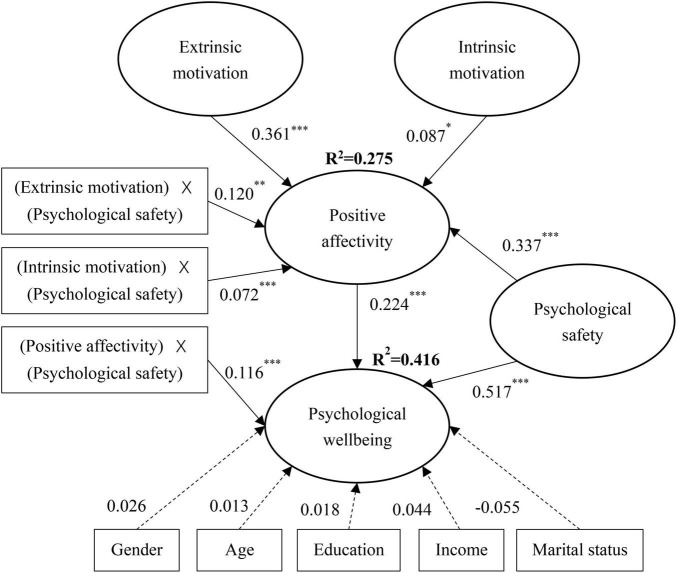
Hypothesis testing. *n* = 391, *p < 0.05, ^**^p < 0.01, ^***^p < 0.001.

An SEM was conducted to test the hypotheses in this study. Results of hypothesis testing are presented in Figure 2. It is indicated that extrinsic motivation was positively related to positive affectivity (β = 0.361, *p* < 0.001), which was positively related to psychological wellbeing (β = 0.224, *p* < 0.01). The bootstrap analysis with 5,000 samples and 95% confidence interval (CI) showed that the indirect effect of extrinsic motivation on psychological wellbeing through positive affectivity was positively significant: β = 0.019, *p* < 0.01, 95% CI = [0.012, 0.056]. Thus, hypothesis H1 was supported.

Similarly, intrinsic motivation was positively related to positive affectivity (β = 0.087, *p* < 0.05), which was positively related to psychological wellbeing (β = 0.224, *p* < 0.01). Bootstrap analysis also showed that the indirect effect of intrinsic motivation on psychological wellbeing through positive affectivity was positively significant: β = 0.079, *p* < 0.01, 95% CI = [0.043, 0.130]. Thus, hypothesis H2 was supported.

In addition, results in Figure 2 indicate that the interaction term between extrinsic motivation and psychological safety (β = 0.120, *p* < 0.01) and that between intrinsic motivation and psychological safety (β = 0.072, *p* < 0.001) were both positively related to positive affectivity. Thus, hypotheses H3 and H4 were supported.

Furthermore, the interaction term between positive affectivity and psychological safety was positively related to psychological wellbeing (β = 0.116, *p* < 0.001). Thus, hypothesis H5 was supported.

## Discussion and Conclusion

### Main Findings and Theoretical Implications

Several antecedents of employees’ psychological wellbeing have been identified in prior research. Unfortunately, the role of individual factors, such as motivations and emotions, has been largely ignored in prior literature. This study found the mediating effect of positive affectivity in the relationship between extrinsic motivation and accountant employees’ psychological wellbeing and between intrinsic motivation and accountant employees’ psychological wellbeing. This finding indicates that extrinsic motivation acts as an incentive mechanism that triggers accountant employees’ feelings of satisfaction and enthusiasm. Consequently, they experience a good psychological wellbeing because their psychological needs and demands are satisfied. Similarly, when accountant employees have strong internal needs, such as self-achievement and skill development, they are motivated by these inner needs that encourage the pursuits for personal growth and development, autonomy, and competence. This intrinsic motivation makes employees feel happy, interested, and satisfied. Psychological wellbeing is more likely a result of this positive emotion and intrinsic motivation. Our findings provide support and extend prior studies, such as Kuvaas et al. (2017) and Dang and Chou (2019), who suggested the important roles of self-determination motivations in influencing employees’ emotions, behavioral outcomes, and psychological wellbeing in the workplace. Thus, this study extends the self-determination theory and helps to clarify the relationship among extrinsic motivation, intrinsic motivation, positive affectivity, and psychological wellbeing. Our findings provide an initial foundation for future researchers who are studying the roles of motivations and emotions in affecting employees’ psychological wellbeing.

In addition, this study found that psychological safety had an influent effect on employees’ motivations, emotions, and behaviors. In particular, it is found that psychological safety moderated the link between self-determination motivations and positive affectivity and between positive affectivity and psychological wellbeing. These results imply that when accountant employees have a perception of safety and security in their organizations, they tend to have strong motivations and experience a positive emotional state and psychological wellbeing because feelings of safety and security act as a mechanism that provides protection to boost employees’ confidence, motivations, and emotions at work. In contrast, if accountant employees have a low level of psychological safety, they are more likely to have less motivations and experience a negative emotional state and less likely to satisfy with their jobs and personal life. The reason is that perception of unsafety, risk, and uncertainty exerts pressure and stress that put employees in a state of psychological depression. The findings of this study are consistent with Edmondson (1999), Das and Varshneya (2017), Newman et al. (2017), and Dang et al. (2021) who claimed and reported the important role of psychological safety in influencing an individual’s motivations, emotions, and behaviors. Thus, the findings of this study shed new light on the moderating mechanism of psychological safety on the relationship between accountant employees’ motivations, emotions, and psychological wellbeing. These findings provide implications for researchers who are investigating the role of psychological safety in affecting employees’ psychological wellbeing in the workplace.

Furthermore, employees’ psychological wellbeing has become an important issue that receives a great attention of researchers and business managers in the past decades (Kundi et al., 2020). In the workplace, improving employees’ psychological wellbeing not only benefits employees’ health conditions and work performance but also increases firms’ productivity and performance (Boudrias et al., 2021). This study proposes and tests a unique model to explain the mediating and moderating mechanisms of positive affectivity and psychological safety in the link between self-determination motivations and psychological wellbeing. Given the absence of the current literature about the influence of these variables on employees’ psychological wellbeing, this study can be viewed as the first in its kind to examine the psychological wellbeing of accountant employees in an emerging country (i.e., China). Therefore, our findings provide implications for researchers who are investigating the issue of employees’ psychological wellbeing in the workplace in emerging countries.

### Practical Implications

This study provides implications for business managers. First, the work of an accountant employee is often stressful and requires high efforts (Pradipta and Bernawati, 2019). Given the importance of psychological wellbeing in the workplace, it is suggested that business managers should care and improve the psychological wellbeing of accountant employees. For example, business managers should have a strategy to enhance the meaningfulness of accountant work, reduce stress and pressure for employees, and improve accountant employees’ motivations and emotions at work.

Second, based on the findings in this study, it is suggested that extrinsic motivation plays an important role in enhancing accountant employees’ positive affectivity and psychological wellbeing. Thus, organizations and business managers should have policies and strategies to provide extrinsic incentives for accountant employees. For example, managers should adopt various extrinsic incentives, such as monetary rewards, compensation, promotion, or non-financial incentives, to induce employees’ positive emotions and feelings of wellbeing.

Third, along with providing extrinsic incentives, organizations and business managers should also have a strategy to improve and enhance accountant employees’ intrinsic motivation. For example, managers should encourage and trigger employees’ inner needs, improve and build an interesting working environment, enhance the enjoyment and pleasure of the workplace, and improve employees’ personal growth and skill development. Such internal needs can induce positive affectivity and psychological wellbeing. A good strategy that effectively utilizes intrinsic motivation can lead to positive affectivity and feelings of psychological wellbeing for employees.

Finally, as indicated in the findings of this study, it is suggested that business managers should have strategy and policy to improve accountant employees’ perception of safety. For example, managers should provide more physical and emotional supports for employees, secure and protect employees’ jobs, commit to the development and growth of employees, and help employees to overcome difficulties at the workplace and in their personal life. A good strategy and policy to raise employees’ perception of psychological safety will enhance their motivations, positive affectivity, and psychological wellbeing.

### Limitations and Future Research

Several limitations exist in this study and need to be addressed in future research. A survey questionnaire has been widely used in the current literature to test the hypotheses, but it has its own limitation in testing causal relationships between variables. Thus, it is suggested that future research should use other methods, such as experiments to test causal relationships. In addition, the sample data in this study were accountant employees in Chinese firms. The results may have limitations in generalizability. Thus, it is suggested that future research should collect data from employees in different countries to validate the research model in this study. Furthermore, this study investigated only the roles of self-determination theory, positive affectivity, and psychological safety, and several other variables may play a role in predicting employees’ psychological wellbeing, such as environmental factors, organizational factors, team factors, leadership factors, and individual factors. These variables should be determined in future research.

## Data Availability Statement

The raw data supporting the conclusions of this article will be made available by the authors, without undue reservation.

## Ethics Statement

Ethical review and approval was not required for the study on human participants in accordance with the local legislation and institutional requirements. Written informed consent from the patients/participants was not required to participate in this study in accordance with the national legislation and the institutional requirements.

## Author Contributions

PL and QW contributed to the editing and analysis of the research. TV and VN contributed to the writing and formatting of the research. All authors contributed to the article and approved the submitted version.

## Conflict of Interest

The authors declare that the research was conducted in the absence of any commercial or financial relationships that could be construed as a potential conflict of interest.

## Publisher’s Note

All claims expressed in this article are solely those of the authors and do not necessarily represent those of their affiliated organizations, or those of the publisher, the editors and the reviewers. Any product that may be evaluated in this article, or claim that may be made by its manufacturer, is not guaranteed or endorsed by the publisher.
